# Evaluation of therapeutic efficacy in hepatocellular carcinoma ablation based on CT radiomics and deep learning multimodal models

**DOI:** 10.3389/fonc.2026.1764094

**Published:** 2026-04-10

**Authors:** Manxia Zhou, Lin Zhu, Kaimin Zhang, Shanshan Wei, Haolun Wang, Songqing He, Xu Liu, Xialing Huang

**Affiliations:** 1 Department of Radiology, The First Affiliated Hospital of Guangxi Medical University, Nanning, Guangxi, China; 2 Guangxi Key Laboratory of Special Biomedicine, School of Medicine, Guangxi University, Nanning, Guangxi, China; 3 Department of Emergency, The Second Affiliated Hospital of Guangxi Medical University, Nanning, Guangxi, China; 4 Clinical Physician Training Base, Guangxi Academy of Medical Sciences and the People’s Hospital of Guangxi Zhuang Autonomous Region, Nanning, Guangxi, China; 5 Key Laboratory of Early Prevention and Treatment for Regional High Frequency Tumor (Guangxi Medical University), Ministry of Education, Nanning, Guangxi, China

**Keywords:** ablation, complete response, deep learning, hepatocellular carcinoma, radiomics

## Abstract

**Objective:**

This study aims to develop a multimodal model that integrates clinical features and preoperative imaging characteristics to predict complete response (CR) in hepatocellular carcinoma (HCC) patients following ablation therapy, and to assess the therapeutic efficacy of ablation therapy.

**Methods:**

From October 2017 to June 2024, we collected clinical data and CT enhanced images from 108 HCC patients within one month before ablation therapy. The most important features were selected and dimensionality was reduced using the Mann-Whitney U test, Principal Component Analysis (PCA), and Least Absolute Shrinkage and Selection Operator (LASSO) regression. A multimodal feature set was constructed by integrating clinical characteristics. The dataset is randomly divided into training and validation sets at a 7:3 ratio. Using 10-fold cross-validation, we employ eight radiomic and deep learning algorithms (such as logistic regression, random forest, and MLP) to train models for predicting the CR of HCC. The performance of the optimal model is then comprehensively evaluated.

**Results:**

A total of 14 clinical datasets were collected, resulting in the extraction of 3,192 radiomic features and 256 deep learning features. Among the clinical datasets, a significant difference was found in the rate of HBV positivity (p<0.05). From the radiomic features, 26 key features were selected and dimensionally reduced, while 5 key features were selected and reduced from the deep learning features. The multimodal feature set (Radiomics + Clinical + Deeplearning) achieved the best AUC results across MLP and several other machine learning models. Notably, the MLP model delivered the highest overall performance, with an AUC of 0.933 in the test set. The accuracy, specificity, and positive predictive value of the MLP model were 0.79, 0.99, and 0.89, respectively.

**Conclusion:**

This pilot study established a multimodal model combining radiomics and deep learning to predict the response to ablation therapy in HCC. The model demonstrated robust performance, providing a reliable tool for personalized efficacy assessment and individualized treatment strategies.

## 1 Introduction

Hepatocellular carcinoma (HCC) is the sixth most common malignant tumor worldwide and the third leading cause of cancer-related deaths. In China, the 5-year survival rate for HCC patients is only approximately 12%-18%. According to the World Health Organization, over 865,000 new cases of HCC were reported worldwide in 2022, with more than 757,000 deaths attributed to the disease, making it the second leading cause of death among men due to cancer (1). Currently, the first-line treatment options for early-stage HCC patients (tumor diameter < 2–3 cm) primarily include surgical resection, liver transplantation, radiofrequency ablation (RFA), and microwave ablation (MWA). However, for patients with limited liver function reserve, liver donor shortage, or tumor location restrictions, liver resection and liver transplantation are not the primary treatment choices (2, 3). In this context, local ablation therapies represented by radiofrequency ablation (RFA) and microwave ablation (MWA) have emerged as first-line treatment options for early-stage HCC due to their minimally invasive nature, repeatability, and minimal impact on liver function (4). However, clinical practice has shown that even with strict adherence to technical guidelines, up to 60% of patients experience early recurrence (5, 6), leading to an increased rate of secondary interventions and worsened prognosis. Therefore, accurately predicting the likelihood of complete response (CR) to ablation therapy preoperatively has become a key scientific issue for optimizing personalized treatment decisions and therapeutic outcomes. Currently, the prediction of the effectiveness of ablation therapy for HCC in clinical practice primarily relies on several factors, including the patient’s liver function status (Child-Pugh classification), overall health status (ECOG performance status), tumor morphological characteristics (such as diameter, number, and location), biological markers (AFP, TP53), morphological features observed in enhanced CT/MRI (such as margins and enhancement degree), and technical operational factors (7–9). However, these parameters have significant limitations. Morphological assessments fail to reflect the internal heterogeneity of tumors, and the sensitivity and specificity of serum biomarkers are insufficient, often being influenced by the background of liver cirrhosis (10). Existing predictive models, such as Support Vector Machine (SVM) models and the Barcelona Clinic Liver Cancer (BCLC) staging system, are primarily constructed based on retrospective cohorts, with their external validation AUC (Area Under Curve) values generally falling below 0.75 (11, 12). This inadequacy makes it challenging to meet the demands of precision medicine.

In recent years, radiomics studies based on CT or MRI have been increasingly applied to the evaluation of therapeutic efficacy in HCC, metastatic tumors, pulmonary nodules and other conditions (11–13), particularly for assessing the efficacy of local treatments for HCC, with promising results (12, 14, 15). Radiomics primarily involves the manual delineation of regions of interest (ROIs) to extract hundreds of quantitative features related to texture, shape, and intensity from CT/MRI images, providing a comprehensive reflection of the internal heterogeneity of tumors (16, 17).

However, for radiomics, the manual delineation of the region of interest (ROI) often leads to poor reproducibility and high feature redundancy. Unlike the manual extraction of radiomic features, deep learning can automatically learn hierarchical abstract features from images to capture tumor edge ambiguity, heterogeneity distribution and other patterns, thus avoiding the micro-patterns that are often unrecognizable to human vision (18, 19). Moreover, deep learning can predict progression-free survival after liver resection without the need for contour delineation; however, it requires substantial data support (20). In the prediction of early recurrence after HCC ablation based on contrast-enhanced ultrasound radiomics, the combined model of radiomics and deep learning achieved an AUC of 0.819, significantly higher than that of individual models (21). Similarly, in the prediction of early recurrence after HCC ablation based on MRI radiomics, the combined model reached an AUC of 0.706, also significantly surpassing that of single models (22). By integrating both approaches in feature extraction, model construction, and outcome prediction, predictive performance can be enhanced.

The “multimodal fusion” strategy, which integrates clinical parameters, radiomic features, and deep learning characteristics, is considered a key direction for overcoming the limitations of single data sources. Huang et al. demonstrated that combining clinical parameters, radiomics, and deep learning features into a multimodal model more effectively distinguished between non-invasive and invasive lung adenocarcinoma, resulting in improved performance of the multimodal model (23). Clinical parameters provide macro-level insights into tumor biological behavior, while radiomic features reflect the spatial distribution of intratumoral heterogeneity, and deep learning features capture sub-visual texture details. Together, these three elements form a complementary information network. For in-stance, Zhang’s team combined serum AFP levels with MRI radiomic and deep learning features to predict postoperative recurrence of HCC, enhancing the AUC from 0.78 for single-modality models to 0.87 (24). How-ever, existing studies are often limited to bimodal combinations (such as Clinical + Radiomics) and lack systematic evaluations of the contributions of different modalities to predictive performance. Currently, there is a scarcity of research exploring the construction of predictive models for recurrence after ablation therapy.

This study aims to explore whether a multi-modal radiomics model based on preoperative CT imaging can be used to predict CR after ablation treatment for HCC. Additionally, it will evaluate the predictive performance of the model under different feature combinations, providing a reliable decision support tool for the efficacy assessment of HCC ablation therapy.

## 2 Methods

### 2.1 Study subjects

A total of 108 patients diagnosed with hepatocellular carcinoma (HCC) between October 2017 and June 2024, who were hospitalized at our institution and underwent their first curative ablation treatment, including microwave ablation (MWA) and radiofrequency ablation (RFA), were included in the study. The enrolled patients had to meet the following criteria: (1) patients classified as category 5 according to the Liver Imaging Reporting and Data System (LI-RADS), and/or patients confirmed to have HCC through pathological biopsy; (2) patients staged as stage I according to the Chinese Liver Cancer Staging(CNLC) system; (3) patients with untreated HCC who were undergoing RFA or MWA for the first time; (4) patients who underwent a non-contrast and contrast-enhanced upper abdominal CT scan within one month prior to treatment; (5) patients who had a follow-up non-contrast and contrast-enhanced upper abdominal CT scan within 1 to 12 months post-treatment and regular follow-up assessments, with efficacy evaluated using the modified Response Evaluation Criteria in Solid Tumors (mRECIST) criteria. Exclusion criteria included: (1) patients classified as categories other than 5 according to LI-RADS, and/or patients confirmed to have intrahepatic cholangiocarcinoma or mixed-type liver cancer through pathological biopsy; (2) patients with CT imaging showing poorly defined diffuse liver cancer; (3) patients who had previously undergone tumor treatments such as transarterial chemoembolization (TACE), hepatic arterial infusion chemotherapy (HAIC), targeted immunotherapy, or radiotherapy.

### 2.2 Clinical characteristics

We retrospectively collected the following basic clinical characteristics of the study patients: age, sex, positivity for hepatitis B virus surface antigen (HBsAg), abnormal prothrombin (Protein Induced by Vitamin K Absence or Antagonist-II, PIVKA-II), alpha-fetoprotein (AFP) levels, and the general health status score of liver cancer patients (performance status score, PS score).

### 2.3 Assessment of tumor response

**Figure 1:**
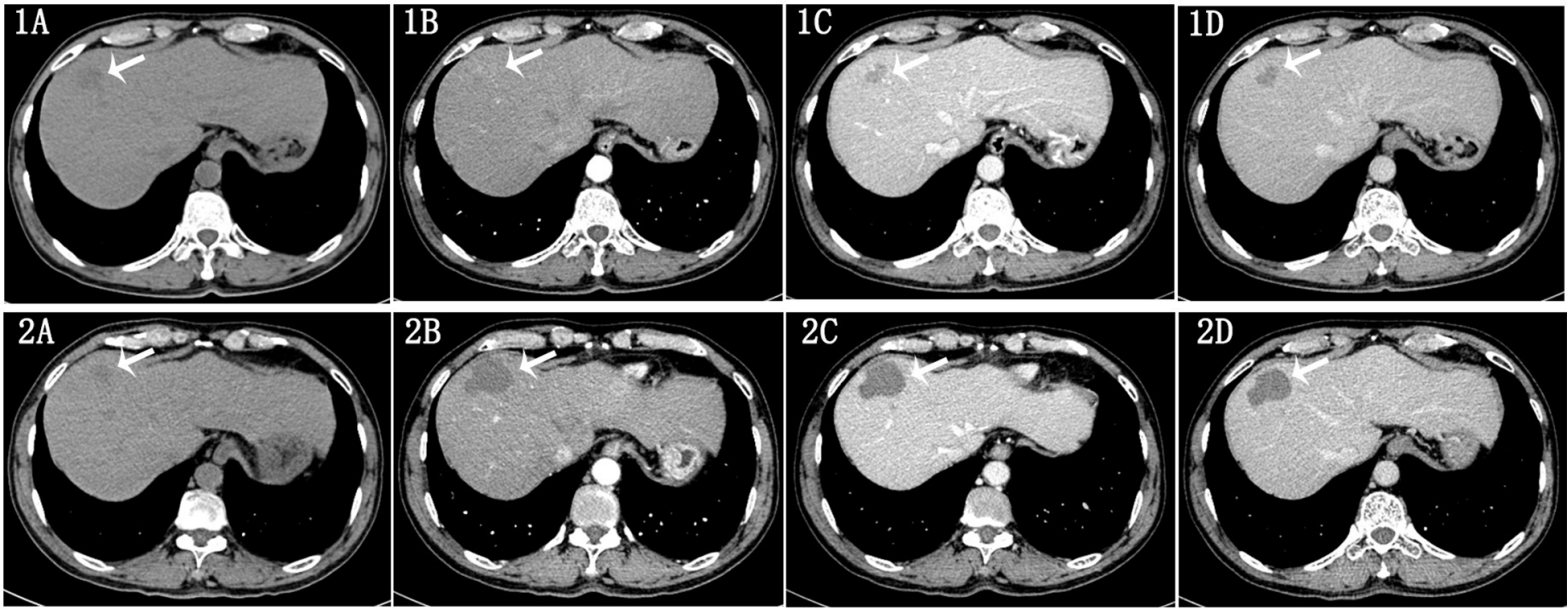
Male, 60 years old, diagnosed with hepatocellular carcinoma (HCC) in liver segment S8 via clinical/pathological biopsy. **(A–D)** show contrast-enhanced CT scans performed 1 week before ablation therapy. **(A)** Non-contrast scan shows a hypodense lesion. **(B)** Arterial phase reveals mild heterogeneous enhancement of the lesion. **(C)** Venous phase demonstrates washout of contrast. **(D)** Delay phase shows further decrease in enhancement. Images 2A–D show follow-up contrast-enhanced CT scans 29 weeks post-ablation therapy: Image 2A: Non-contrast scan shows a hypodense lesion. Image 2B: Arterial phase shows no significant enhancement. Images 2C, D: Venous and delayed phases confirm persistent absence of enhancement. Conclusion: Complete resolution of the lesion after ablation therapy.

**Figure 2:**
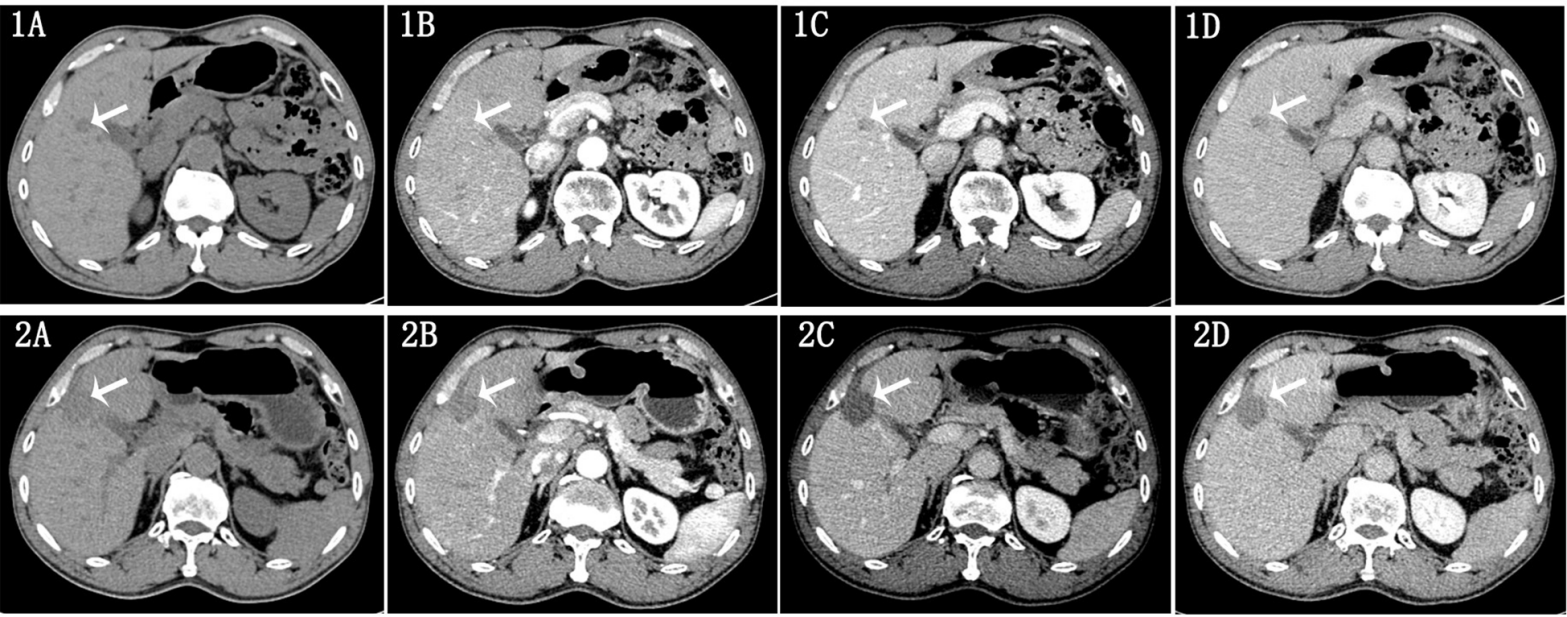
Male, 58 years old, diagnosed with HCC in liver segments S4/5 via clinical/pathological biopsy. Images 1A–D show contrast-enhanced CT scans 1 week before ablation therapy: Image 1A: Non-contrast scan shows a hypodense lesion. Image 1B: Arterial phase reveals mild heterogeneous enhancement. Image 1C: Venous phase demonstrates washout of contrast. Image 1D: Delayed phase shows further decrease in enhancement. **(A–D)** show follow-up contrast-enhanced CT scans 18 weeks post-ablation therapy: **(A)** Non-contrast scan shows a hypodense lesion. **(B)** Arterial phase reveals mild peripheral enhancement. **(C, D)** Venous and delayed phases show decreased enhancement. Conclusion: Incomplete resolution of the lesion after ablation therapy.

The assessment of treatment response was conducted by radiologists with 5 and 16 years of experience in abdominal imaging, respectively, based on the modified Response Evaluation Criteria in Solid Tumors (mRECIST) for HCC (25). The evaluation of enhanced CT images and clinical manifestations before and after curative ablation treatment was categorized as follows: (1) Complete Response (CR): Disappearance of arterial enhancement in all liver lesions; (2) Partial Response (PR): A reduction of ≥50% in the sum of the diameters of the target lesions’ arterial enhancement; (3) Progressive Disease (PD): An increase of ≥25% in the sum of the diameters of the target lesions’ arterial enhancement or the appearance of new lesions; (4) Stable Disease (SD): Lesions that do not meet the criteria for PR or PD. We defined CR as the complete response group (**Figure 1**), while PR, SD, and PD were classified as the non-complete response group (**Figure 2**). In cases of disagreement between the two radiologists, a consensus was reached through discussion.

### 2.4 CT image acquisition

The scanning parameters of Siemens du-al-source spiral CT (SOMATOM Definition Flash, Germany) were: thickness of 1.5 mm, pitch of 0.8, tube voltage of 120 kV, and tube current of 210 mAs. The scanning parameters of GE 64-slice spiral CT scanner (Light Speed VCT, Germany) were: thick-ness of 1.25 mm, pitch of 0.984, tube volt-age of 100 kV, tube current of 400 mAs. First, the patients underwent a scan of the whole liver, followed by enhanced scanning. A contrast agent was used in the imaging sessions (Iopromide, 300 mgI/mL, Germany Bayer Healthcare Co.), with a dosage of 1.5 mL/kg and blood flow rate of 3 mL/s through the superficial vein on the elbow with a double-barrel high-pressure syringe. At 30 s, 60 s, and 120 s post-injection, the arterial phase, portal vein phase, and delayed phase were scanned, respectively. To minimize scanner-related variability, all images underwent standardized preprocessing: (1) resampling to 1.0 mm isotropic voxels using B-spline interpolation; (2) application of fixed window settings (width 350 HU, level 40 HU) for consistent liver contrast; (3) Z-score normalization to account for inter-scanner intensity variations.

### 2.5 Evaluation of imaging features

Preoperative enhanced CT imaging features were observed, including tumor number, lo-cation, margins, morphology, degree of arterial phase enhancement, portal phase washout, and delayed phase encapsulation of the tumor. The images were assessed by radiologists with 5 and 16 years of experience in abdominal imaging, respectively. In cases of disagreement, a consensus was reached through discussion.

### 2.6 Image segmentation

**Figure 3:**
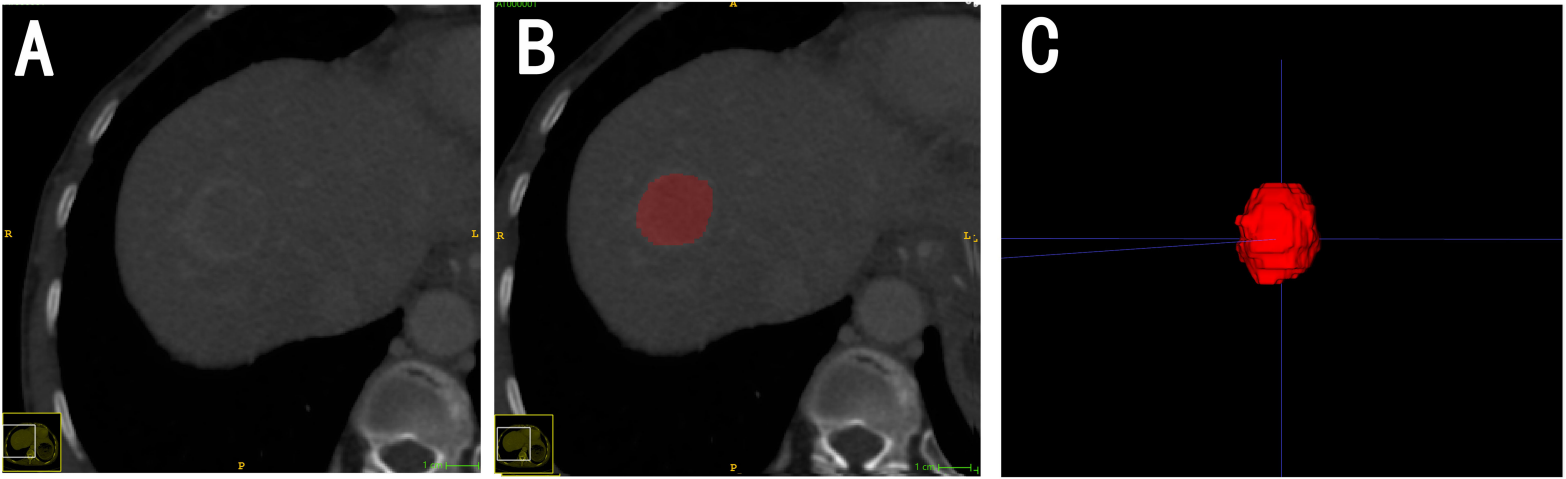
An example of the manual segmentaion in hepatocelular carcinoma. **(A)** Venous phase. **(B)** Manualsegmentation on the same axial slice. **(C)** Generation of a 3D ROl.

Arterial phase, portal phase, and delayed phase CT images were retrieved from the Picture Archiving and Communication System (PACS) for tumor segmentation. The DICOM images from the three phases were loaded into ITK-SNAP software (26) (ver-sion4.2.0, http://www.itksnap.org) for manual segmentation. During the segmentation process, efforts were made to closely outline the tumor margins, generating three-dimensional volumes of interest (VOI). The images were delineated by radiologists with 5 and16 years of experience in abdominal imaging, respectively, ensuring coverage of the entire tumor (**Figure 3**). Results with a consistency greater than 0.7 were selected for further analysis.

### 2.7 Radiomic feature extraction and dimensionality reduction

We utilized the Pyradiomics module (https://github.com/Radiomics/pyradiomics) for feature extraction. The extracted radio-mic features were categorized into four groups: I. Gray Level Features, II. Shape Features, III. Texture Features, and IV. Wavelet Features. A total of 3,192 three-dimensional (3D) radiomic features were obtained. To select the most valuable radiomic features for predicting CR, we first applied feature scaling to reduce the variability among the features. Significant features were screened using the Mann-Whitney U test, with a p-value of <0.05 indicating a statistically significant difference. To extract the intrinsic structure of the data, reduce redundancy, and minimize errors caused by noise, we performed dimensionality reduction using Principal Component Analysis (PCA) to synthesize new features. Additionally, we applied the Least Absolute Shrinkage and Selection Operator (Lasso) in a logistic regression model, setting 200 equally spaced values within the range of (10^-6, 10^1). The optimal alpha value was determined using the Mean Squared Error (MSE) metric, allowing us to filter out features that were highly correlated with CR from the initial 266 features.

### 2.8 Deep learning features and dimensionality reduction

Deep learning models were trained using Convolutional Neural Networks (CNN) and Transformers. Initially, the imaging data underwent preprocessing, in which the raw CT images were inputted, and the pixel values were normalized to a range of 0-1000. Images containing lesions were cropped to a size of 170×113×46 pixels based on the maximum dimensions of the labels in the entire dataset. Due to significant differences in data range, standard deviation, and mean, Z-score normalization was applied to eliminate numerical biases between different samples. Subsequently, a four-dimensional data structure (3, 170, 113, 46) was established using the three phases: “arterial phase”, “portal phase” and “delayed phase”, where (170, 113, 46) represents the original dimensions of the data. Feature extraction was performed on the input data, yielding 128 features from both the 3D ResNet50 and the Vision Transformer, resulting in a total of 256 features was established using the three phases: “arterial phase”, “portal phase” and “delayed phase”, where (170, 113, 46) represents the original dimensions of the data. Feature extraction was per-formed on the input data, yielding 128 features from both the 3D ResNet50 and the Vision Transformer, resulting in a total of 256 features. We employed the Wilcoxon rank-sum test to analyze the differences between the two groups, selecting features with statistically significant differences.

### 2.9 Model construction and evaluation

**Figure 4:**
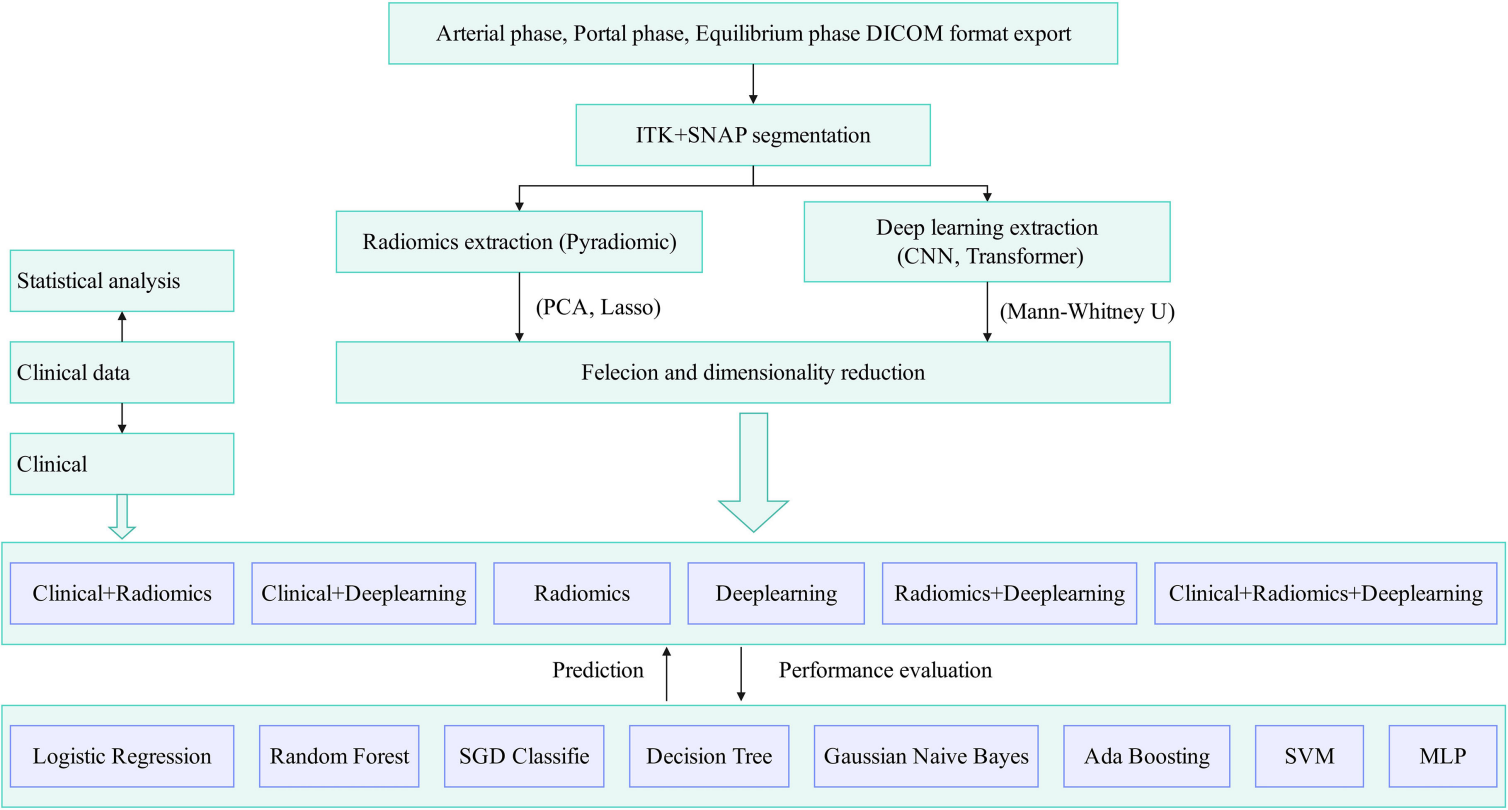
Flowchart of the study. (PCA, principal component analysis; LASSO, least absolute shrinkage and selection operator; M-W, Mann-Whitney U test; SVM, support vector machine ; MLP, multilayer perceptron).

We utilized the Scikit-learn package (version 0.18) in Python 3.7 for model construction and evaluation. Our dataset was randomly divided into a training set (n=75) and a testing set (n=33) in a 7:3 ratio. The training set was used for model construction, while the testing set was employed for model evaluation. We created combinations of clinical features, radiomic features, and deep learning features, categorized as follows: A (Radiomics), B (Deeplearning), C (Radiomics + Clinical), D (Deeplearning + Clinical), E (Radiomics + Deeplearning), and F (Radiomics + Clinical + Deeplearning). We then applied eight robust classification algorithms, including Logistic Regression, Random Forest, SGD Classifier, Decision Tree, Gaussian Naive Bayes, Ada Boosting, Sup-port Vector Machine (SVM), and Multi-Layer Perceptron (MLP), using an internal ten-fold cross-validation grid search method to calculate the Area Under the Curve (AUC) for each feature set. The feature set with the highest predictive capability was used to establish the optimal model, and the model’s predictive performance was visually represented through the Receiver Operating Characteristic (ROC) curve. The research flowchart is shown in **Figure 4**.

### 2.10 Decision curve analysis

We performed decision curve analysis to assess the clinical utility of our prediction models, DCA was performed using the rmda package in R software (version 4.2.0).

### 2.11 Statistical analysis

Baseline data for patients were expressed as mean ± standard deviation for continuous variables. Clinical characteristics and imaging features associated with CR were analyzed using the Chi-square test in SPSS (version 20.0). Factors with a p-value less than 0.05 were considered to have statistically significant differences. Feature selection and modeling were conducted using Python software (version 3.7). The model generated ROC curves and calculated the AUC Model performance was evaluated using accuracy, sensitivity, specificity, positive predictive value, negative predictive value, and F1 score. The optimal model was selected using the DeLong test.

## 3 Results

### 3.1 Clinical information and tumor response assessment of patients

A total of 108 eligible HCC patients were included in this study, comprising 93 males and 15 females, with ages ranging from 35 to 79 years (mean age 54.36 ± 11.47 years). All tumors had a diameter of less than 3 cm. Based on the assessment of treatment response following tumor ablation, patients were categorized into a complete response group (n=85, 78.70%) and a non-complete response group (n=23, 21.30%). Among the clinical information, only HBV status showed a statistically significant difference between the two groups (P < 0.05). There were no significant statistical differences in patient gender, age, tumor location, margins, morphology, arterial phase tumor enhancement, portal phase tumor washout, equilibrium phase tumor encapsulation, methods of curative ablation treatment, or clinical characteristics such as APT and AFP levels (**Table 1**).

**Table 1:** Clinical and imaging characteristics of patients.

Name	CR (n=85)	Non-CR (n=23)	Cramer’s V	95% CI^2^	P value
Gender			0.097	-4.249-4.411	0.518
Male	72 (84.71%)	21 (91.30%			
Female	13 (15.29%)	2 (8.70%)			
Age (years)	56.70 ± 8.23	56.78 ± 11.43	0.008	-0.257-0.666	0.975
Ablation			0.041	-0.346-0.576	0.689
MWA	77 (90.59%)	20 (86.96%)			
RFA	8 (9.41%)	3 (13.04%)			
HBsAg			0.279	0.182-1.119	0.008*
Positive	72 (84.71%)	13 (56.52%)			
Negative	13 (15.29%)	10 (43.48%)			
AFP (ng/mL)			0.164	-0.045-0.884	0.750
>8.78	43 (50.59%)	15 (65.22%)			
<8.78	42 (49.41%)	7 (34.78%)			
PIVKA-II			0.178	-0.018-0.911	0.140
Positive	37 (43.53%)	15 (65.22%)			
Negative	48 (56.47%)	8 (34.78%)			
PS-Score			0.063	-0.267-0.656	0.382
0	84 (98.82%)	22 (95.65%)			
1	1 (1.18%)	1 (4.35%)			
Tumor location			0.178	-0.029-0.899	0.111
Left lobe	15 (17.65%)	1 (4.35%)			
Right lobe	70 (82.35%)	22 (95.65%)			
Tumor margins			0.097	-0.224-0.700	0.314
Clear	38 (44.71%)	13 (56.52%)			
Blur	47 (55.29%)	10 (43.48%)			
Tumor morphology			0.003	-0.422-0.499	0.971
regular	75 (88.24%)	20 (86.96%)			
Irregular	10 (11.76%)	3 (13.04%)			
Arterial enhancement			0.097	-0.257-0.666	0.417
Significant	72 (84.71%)	21 (91.30%)			
Mild	13 (15.29%)	2 (8.70%)			
Venous phase washout			0.048	-0.346-0.576	0.837
washout	39 (45.88%)	10 (43.49%)			
Non-washout	46 (54.12%)	13 (56.51%)			
Delayed phase capsule			0.065	-0.305-0.617	0.460
Yes	20 (23.53%)	7 (30.43%)			
None	65 (76.47%)	16 (69.57%)			
Number of tumors			0.105	-0.193-0.732	0.305
1	74 (87.06%)	17 (73.91%)			
>1	11 (12.94%)	5 (26.99%)			

MWA, Microwave Ablation; RFA, Radiofrequency Ablation; AFP, Alpha-fetoprotein; HBsAg, Hepatitis B surface; PIVKA-II, Protein Induced by Vitamin K Absence or Antagonist-II. * indicates a statistically significant difference in the P-value.

### 3.2 Feature extraction and selection

**Figure 5:**
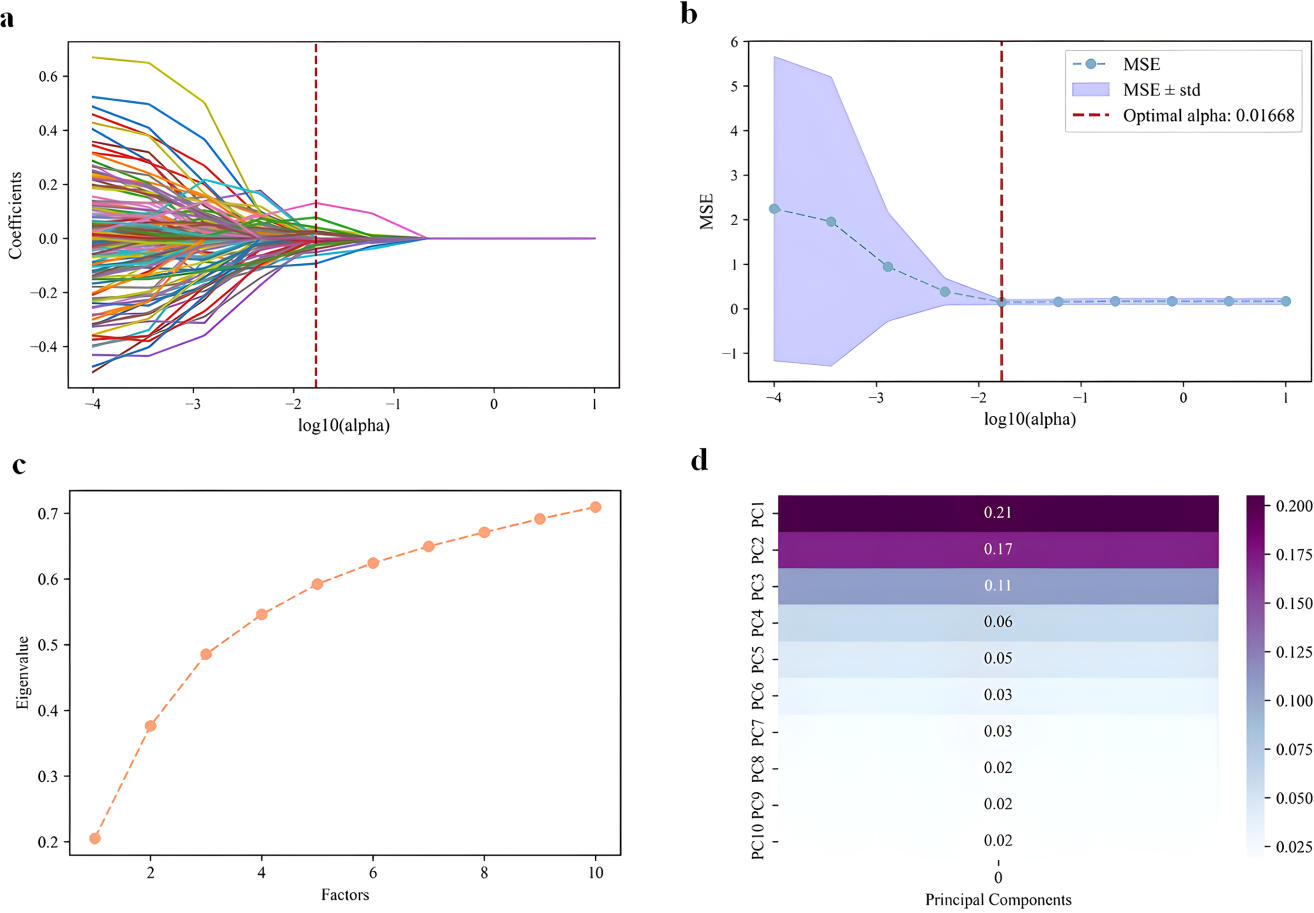
Selection of radiomics and deeplearning features. **(A)** LASSO regression path of radiomics features **(B)** Cross-validation curve of LASSO for radiomics features **(C)** Cumulative explained variance curve of radiomics features **(D)** PCA loadings heatmap of radiomics features.

In this study, a total of 3,192 radiomic features and 256 deep learning features were extracted, followed by a systematic feature selection and dimensionality reduction analysis aimed at predicting CR after ablation treatment for HCC. Among the radiomic features, the Mann-Whitney U test was used to identify 266 features associated with CR following HCC ablation. Subsequently, PCA was employed to reduce the dimensionality of these features, resulting in the generation of 10 new features. Additionally, LASSO logistic regression method was applied to select 16 key features from this set (**Figure 5**), yielding a total of 26 important radiomic features. Regarding the deep learning features, we extracted 256 feature values based on CNN and Transformer architectures. The results of the differential analysis indicated that 5 features were significantly associated with the CR outcome. These key features provide valuable imaging information from the deep learning perspective to enhance the prediction of ablation efficacy in HCC.

### 3.3 Model development and performance

**Figure 6:**
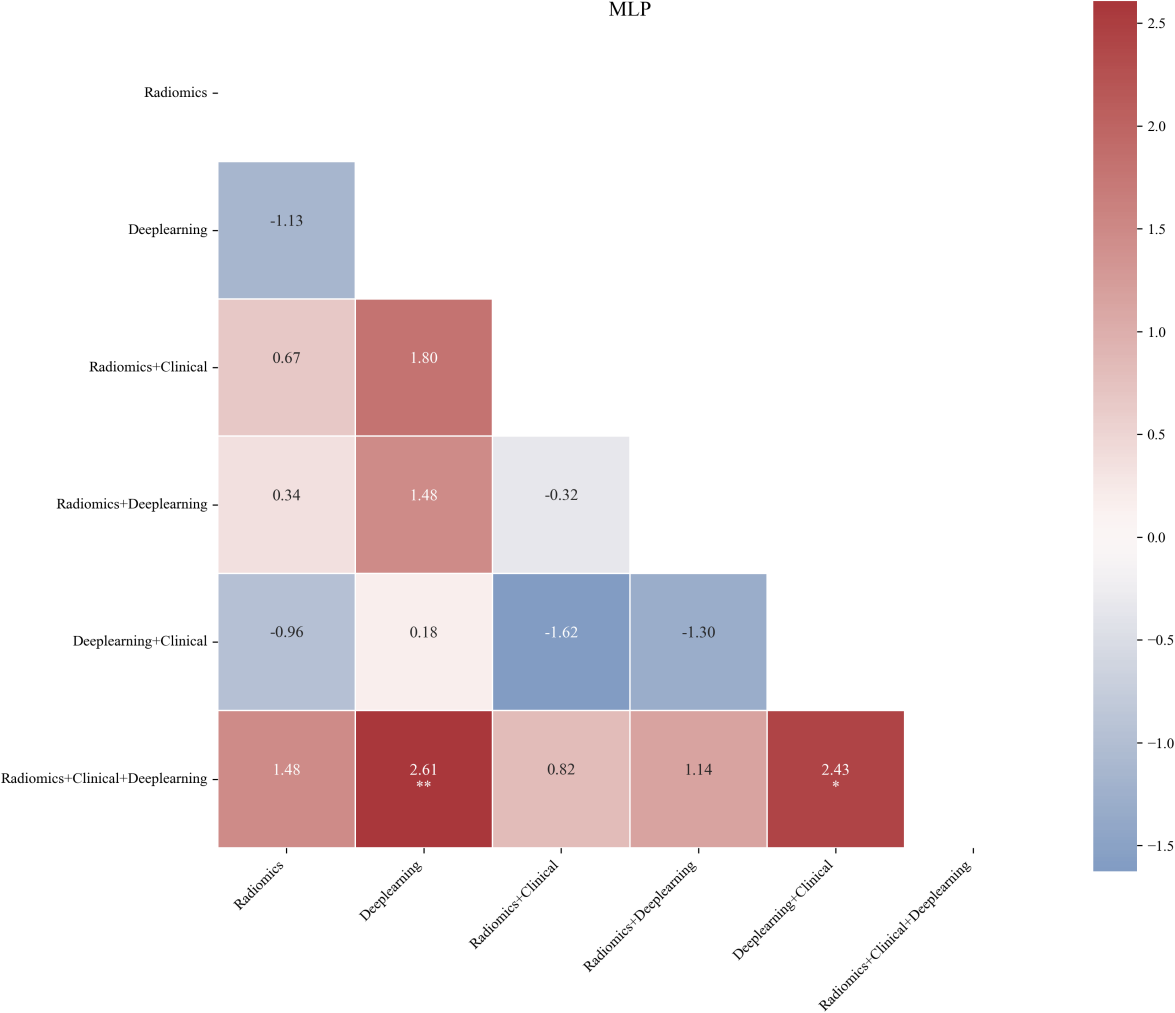
Heatmap of Delong's test. The values in the image represent the Delong test statistic (*Z* value and *D* value), *P<0.05, **P<0.01.

**Figure 7:**
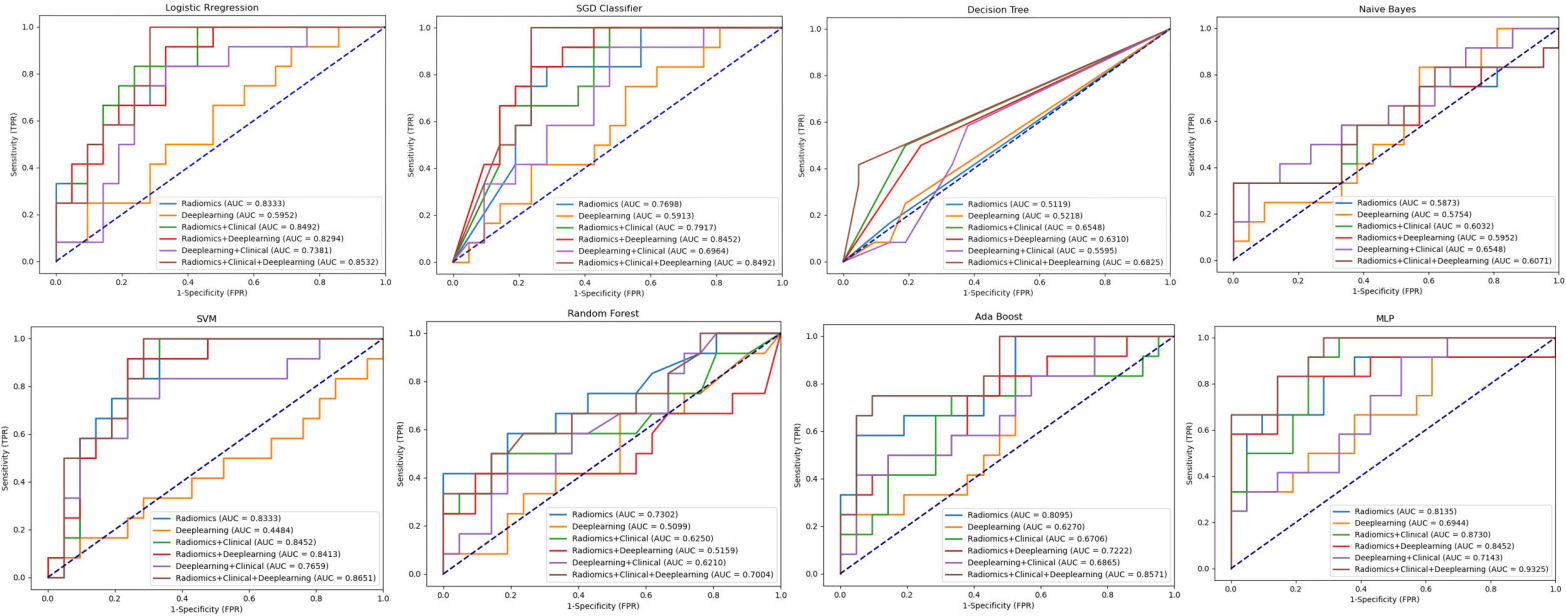
ROC curves of classification algorithm models across different feature combinations and area under the curve (AUC) are reported.

To identify the optimal feature combination, we performed a comprehensive analysis of six established feature combinations, with the AUC as the evaluation metric on the test set. According to the results of the DeLong test (as shown in **Figure 6**, which presents the statistical difference analysis of each combination under the MLP classification algorithm), the combination F(Radiomics + Clinical + Deeplearning) exhibited statistically significant differences when compared to combinations B(Deeplearning) and D(Deeplearning + Clinical) (0.933 vs 0.694, P < 0.01; 0.933 vs 0.845, P < 0.05). The AUC value of combination F(Radiomics + Clinical + Deeplearning) was higher than that of the other combinations A(Radiomics), C(Radiomics + Clinical), and E (Radiomics + Deeplearning); however, these differences were not statistically significant (0.933 vs 0.813, P > 0.05; 0.933 vs 0.873, P > 0.05; 0.933 vs 0.714, P > 0.05). Additionally, we conducted a detailed comparison of the AUC values for each feature set across all models (**Table 2**; **Figure 7**). The F(Radiomics + Clinical + Deeplearning) combination consistently achieved high AUC values in most models, particularly excelling in the MLP (0.9325), Logistic Regression (0.8532), and SVM (0.8651) models, indicating strong discriminative power. Furthermore, combinations C(Radiomics + Clinical) and E (Radiomics + Deeplearning) also demonstrated superior classification performance in certain models, with notably higher AUC values for MLP in combination C(Radiomics + Clinical) (0.873) and combination E(Radiomics + Deeplearning) (0.8452) compared to other models. This suggests that radiomic feature combinations can further enhance classification performance when integrated with clinical factors or deep learning features. In contrast, the overall AUC performance of the deep learning model was relatively low, particularly in the SVM (0.4484), SGD Classifier(0.4563), and Random Forest (0.5099) models. This indicates that relying solely on deep learning features may result in some information deficiency. The AUC values for combination D(Deeplearning + Clinical) exhibited considerable variability across models; although it performed reasonably well in SVM (0.7659) and Logistic Regression (0.7381), it still fell short of the performance of combination F(Radiomics + Clinical + Deeplearning). In summary, considering both the AUC values and the results of the DeLong test, combination F(Radiomics + Clinical + Deeplearning) achieved the best results across the MLP and several other machine learning models. This indicates that this feature combination is capable of more comprehensively integrating information from imaging, clinical, and deep learning features, thereby enhancing the discriminative ability of the models.

**Table 2:** AUC values of different classification models on the test set.

Feature set	Logistic regression	SGD classifier	Decision tree	Gaussian Naive Bayes	SVM	Random forest	Ada boost	MLP
A (Radiomics)	0.8333	0.5556	0.5119	0.5873	0.8333	**0.7302**	0.8095	0.8135
B (Deeplearning)	0.5952	0.4563	0.5218	0.5754	0.4484	0.5099	0.627	0.6944
C (Radiomics+Clinical)	0.8492*	0.5556	0.6548*	0.6032	0.8452*	0.625	0.6706	0.873
D (Radiomics+Deeplearning)	0.7381	0.6409	0.5595	0.6548*	0.7659	0.621	0.6865	0.7143
E (Deeplearning+Clinical)	0.8294	0.6786*	0.631	0.5952	0.8413	0.5159	0.722*	0.845*
F (Radiomics+Clinical+Deeplearning)	**0.8532**	**0.7183**	**0.6825**	**0.6071**	**0.8651**	0.7004*	**0.8571**	**0.9325**

Bold value indicates best result, *is used to denote the runner-up result.

To evaluate the superiority of the established MLP model, we compared it against mainstream machine learning models using the optimal feature combination (Radiomics + Clinical + Deeplearning) across multiple performance metrics. The experimental results are presented in **Table 3**, where the MLP model demonstrated superior performance on several indicators, particularly in accuracy (0.79), precision (0.89), recall (0.75), and F1-score (0.77), outperforming other models. In contrast, traditional machine learning models exhibited certain limitations in performance; for instance, the sensitivity of Logistic Regression, SVM, and AdaBoost was only 0.25, while Decision Tree and Naive Bayes showed weak recall rates. Additionally, although the Random Forest model achieved a specificity of 0.99, its sensitivity was only 0.25, indicating a low capability for recognizing positive samples. Overall, the MLP model maintained a high specificity (0.99) while also improving sensitivity (0.5) compared to other models, and it achieved the best results in terms of precision and F1-score, although this represents the best achievable performance with the current dataset, the sensitivity of 0.5 for non-CR detection may limit the model’s direct clinical utility, as accurately identifying non-responders is critical for timely treatment adjustment. This limitation is primarily attributable to the small number of non-CR cases (n=23) in our cohort.

**Table 3:** Performance of models on the test set with radiomics + clinical + deep learning combined features.

Model	Accuracy	Sensitivity	Specificity	Precision	Recall	F1-score
Logistic Rregression	0.69 (0.55–0.82)	0.25 (0.18–0.45)	0.95 (0.84–0.99)	0.72 (0.53–0.87)	0.6 (0.51–0.70)	0.59 (0.45–0.72)
SGD Classifier	0.7 (0.52–0.88)	**0.58** (0.36–0.76)	0.79 (0.63–0.88)	0.67 (0.56–0.80)	0.67* (0.56–0.80)	0.67* (0.56–0.80)
Decision Tree	0.73* (0.55–0.81)	0.33 (0.18–0.53)	0.95 (0.84–0.99)	0.75 (0.60–0.88)	0.64 (0.54–0.75)	0.64 (0.51–0.77)
Gaussian Naive Bayes	0.67 (0.56-0.78)	0.5 (0.31-0.69)	**0.99** (0.63-0.99)	0.58 (0.52-0.78)	0.58 (0.52-0.76)	0.58 (0 52-0.76)
SVM	0.69 (0.40–0.73)	0.25 (0.21–0.40)	0.95* (0.84–0.99)	0.72 (0.53–0.87)	0.6 (0.55–0.69)	0.59 (0.45–0.72)
Random Forest	0.72 (0.52–0.88)	0.25 (0.17–0.39)	**0.99** (0.91–0.99)	0.85* (0.78–0.91)	0.62 (0.54–0.72)	0.61 (0.47–0.74)
Ada Boost	0.7 (0.57–0.88)	0.25 (0.12–0.45)	0.95 (0.84–0.99)	0.72 (0.50–0.88)	0.6 (0.51–0.70)	0.59 (0.40–0.71)
MLP	**0.79** (0.67–0.94)	0.5* (0.31–0.69)	**0.99** (0.91–0.99)	**0.89** (0.83–0.94)	**0.75** (0.65–0.85)	**0.77** (0.64–0.88)

Bold value indicates best result, *is used to denote the runner-up result.

### 3.4 Clinical utility assessment via decision curve analysis

**Figure 8:**
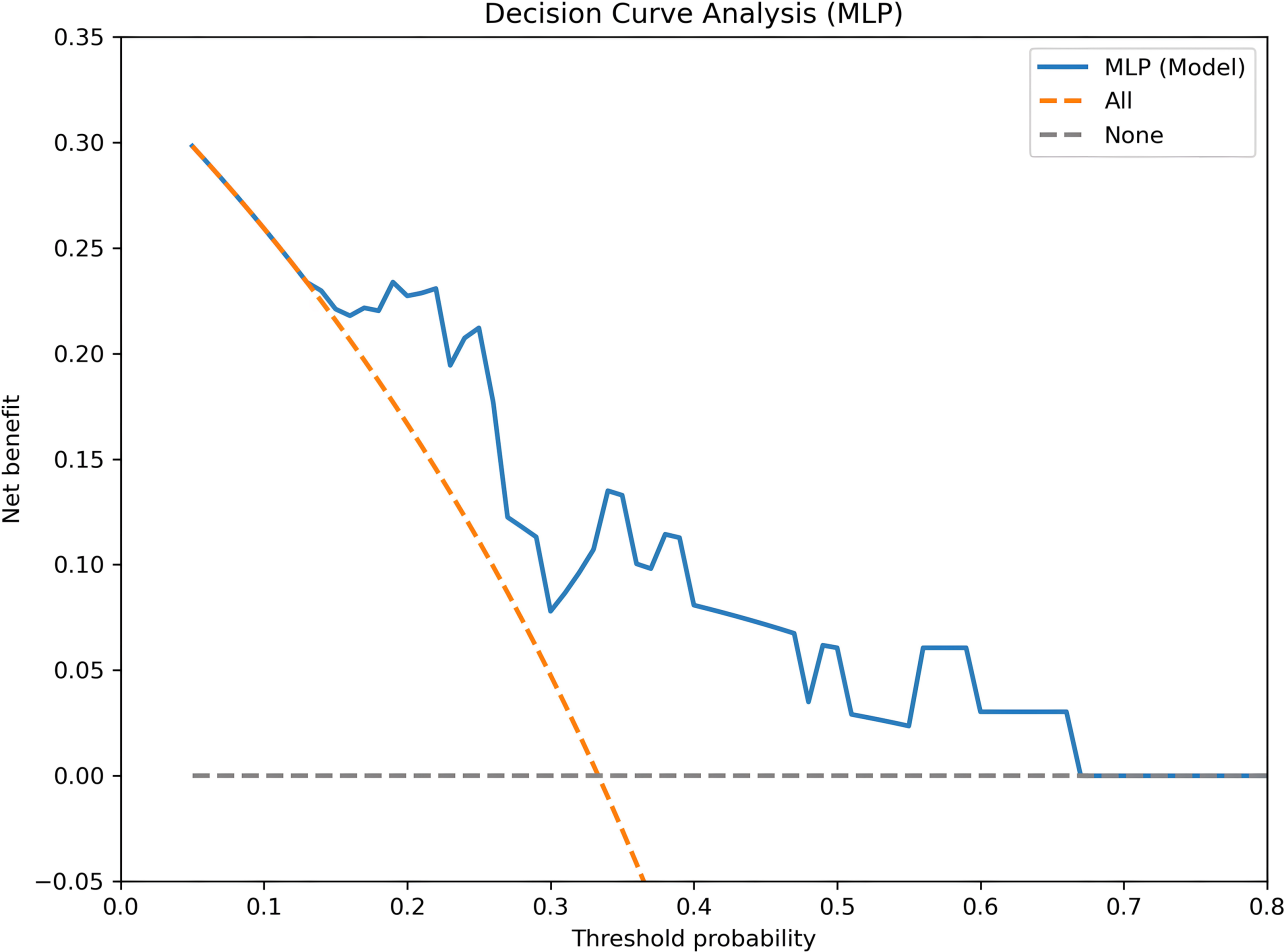
Decision curve analysis for the MLP model with multimodal features (radiomics + clinical + deep learning). The y-axis represents net benefit, and the x-axis represents threshold probability for treatment modification. The blue solid line shows the net benefit of the MLP model. The orange dashed line represents the "treat all" strategy (intervene in all patients), and the grey dashed line represents the "treat none" strategy (intervene in no patients).

To evaluate the potential clinical value of our MLP model, we performed decision curve analysis across a range of clinically relevant threshold probabilities (0 to 60%). As shown in **Figure 8**, the MLP model with multimodal features (Radiomics + Clinical + Deeplearning) demonstrated positive net benefit across threshold probabilities from approximately 15% to 45%, consistently outperforming both the “treat all” and “treat none” strategies. This indicates that using the model to guide treatment decisions within this threshold range would lead to better clinical outcomes compared to default strategies. At the default threshold of 0.5, the model’s net benefit approached zero, consistent with the observed sensitivity of 0.50 for non-CR detection. However, at a lower threshold of 0.3, which might be preferred if clinicians wish to increase sensitivity for identifying non-responders, the net benefit increased substantially, suggesting that a threshold in the range of 0.2–0.4 could optimize clinical utility.

## 4 Discussion

In our study, we found that the clinical characteristic of HBV positivity rate is a significant clinical variable associated with CR following ablation treatment for HCC (P < 0.05). This finding aligns with the epidemiological characteristics of HCC, which is predominantly associated with HBsAg in East Asia (1). The percentage of HBsAg positivity in the non-CR group was relatively low, which we believe may be related to the smaller sample size. It may also suggest that HBsAg-negative patients are less responsive to ablation therapy, a hypothesis that warrants further investigation in future studies. Campani et al. found that the sensitivity and specificity of AFP significantly decline in the context of cirrhosis, particularly performing poorly in early-stage HCC (8). In our study, there was no significant statistical difference in AFP levels between the complete response and non-complete response groups among HCC patients, which may indicate that the underlying liver conditions of the tumors are relatively consistent.

In terms of feature selection, radiomic features were screened using the Mann-Whitney U test, followed by LASSO regression and PCA for dimensionality reduction, ultimately retaining 26 radiomic features. The Mann-Whitney U test is suitable for small sample data, while LASSO regression automatically selects key variables to avoid overfitting. The combination of these two methods enhances model performance and interpretability, accommodating the high-dimensional and small sample characteristics of medical data (10), which aligns with the sample size of this study. For deep learning feature extraction, we first applied Z-Score normalization and resized the data to a uniform dimension, resulting in three-dimensional data. This approach addressed the issues of varying data ranges and standard deviations. We utilized a hybrid model combining Convolutional Neural Networks (CNN) and Transformers, which balances local details (such as tumor edge enhancement) with global dependencies (such as multi-phase enhancement patterns), overcoming the limitations of traditional CNNs in capturing long-range features. As a result, we identified five significantly related features. This study enhanced the discriminative ability of the feature set by integrating radiomic and deep learning features. The combination of clinical, radiomic, and deep learning features achieved favorable results in this research. For instance, Wei et al. utilized MRI radiomics and deep learning to predict early recurrence of HCC, achieving an AUC of 0.706, which improved to 0.762222 upon incorporating clinical features (22). In contrast, the higher AUC of 0.9325 in our study may be attributed to the rich information content of multi-phase CT data, indicating the potential of this method in predicting HCC recurrence.

Accurately assessing CR after curative ablation for HCC is crucial for individualized efficacy evaluation and treatment strategies. By employing an integrated learning approach based on CT radiomics, it is possible to predict the prognosis following curative ablation for HCC. However, there have been few studies reporting the use of a combination of clinical features, radiomics, and deep learning features to predict CR after curative ablation for HCC. In our study, to meet the demands of personalized treatment, we developed a multimodal model based on clinical features, radiomics, and deep learning, utilizing various classification algorithms to predict CR after curative ablation for HCC. Among these, the multimodal model combining clinical features, radiomics, and deep learning achieved the highest AUC value (0.9325) with the MLP classification algorithm, demonstrating a strong ability to predict CR for HCC. This finding aligns with the recent trend in multimodal fusion research. For instance, Huang et al. predicted the invasiveness of lung adenocarcinoma by combining CT imaging with clinical parameters, finding that the AUC of the multimodal model (0.9540) was higher than that of the single-modality AUC (0.6726) (23). Schön et al. established a Cox regression model based on radiomic features and CNN, combined with clinical parameters to predict overall survival (OS), and found that the performance of the multimodal model surpassed that of single models (19). Zhang et al. discovered that a radiomic model incorporating deep learning-based automatic segmentation, circular APHE, and incomplete tumor “capsule” along with clinical parameters such as AFP accurately predicted early postoperative recurrence of individual HCC cases, outperforming the BCLC and CNLC systems (22).

Additionally, we found that the MLP neural network classification algorithm model achieved optimal evaluation metrics. The promising performance of the MLP model in this study may be attributed to its ability to handle high-dimensional data and nonlinear relationships. Deep learning models, such as MLP, can automatically extract complex features through multi-layer network structures, making them particularly suitable for the fusion analysis of radiomic and clinical data (27). Saillard et al. confirmed that MLP can significantly enhance the accuracy of survival predictions by capturing microscopic texture features in the analysis of liver cancer tissue pathology slides (20). Sami et al. found that training the MLP model on nine selected surgical indicators achieved a testing accuracy of 80% (28). El-Sayed et al. utilized MLP to improve lung cancer classification (29). This study further extends these findings, demonstrating that among eight robust classification algorithm models, the MLP outperformed others in accuracy (0.79), sensitivity (0.5), specificity (0.99), positive predictive value (0.89), negative predictive value (0.75), and F1 score (0.77), indicating that MLP also has significant advantages in ablation therapy. This may be due to its strong nonlinear fitting capabilities. Traditional models, such as Logistic Regression (AUC = 0.8532) and SVM (AUC = 0.8651), while performing reasonably well, exhibit limitations when handling high-dimensional, nonlinear data (30). The SGD Classifier showed better sensitivity than MLP; sensitivity measures the model’s ability to correctly identify actual positive class samples (label = 1, non-CR). However, our data included very few non-CR patients(n=23), resulting in poor sensitivity metrics across all models, highlighting the need for future studies to expand the sample size for continued model training. resulting in a sensitivity of only 0.50 for non-CR detection across all models. This level of sensitivity, while representing the best performance among the models tested, may not be sufficient for direct clinical application, where accurately identifying non-responders is essential for timely treatment adjustment (1). The limited number of non-CR cases constrained the model’s ability to learn robust patterns for this clinically critical population, a common challenge in pilot studies (31). Future multi-center collaboration to expand the dataset, particularly enriching for non-CR cases, is essential to improve model sensitivity and generalizability (32, 33).

The choice of threshold should balance false positives (unnecessary interventions) and false negatives (missed non-responders) (34). At the default 0.5 threshold, sensitivity is 0.50 and specificity 0.99. DCA (**Figure 8**) identifies an optimal threshold range of 20-40%, where net benefit is maximized. Within this range, sensitivity would increase to approximately 0.65-0.70, with a modest reduction in specificity (~0.95), better aligning with the clinical priority of identifying non-responders (35). The optimal threshold should be determined through shared decision-making based on clinical context and patient preferences (36).

Given the relatively small sample size (n=108, with 23 non-CR cases), we assessed whether the complexity of our MLP model is justified. Deep learning models risk overfitting with limited data (31); thus, we implemented three mitigation strategies: (1) using pre-trained feature extractors (ResNet50, Vision Transformer) for transfer learning (37); (2) rigorous feature selection reducing 3,192 radiomic features to 26 via LASSO and PCA (38); (3) 10-fold cross-validation during model development (39).To directly address whether the added complexity provides tangible benefits, we compared MLP with the simplest approach: logistic regression using radiomics alone. As shown in **Table 3**, the MLP model with multimodal features achieved significantly higher AUC (0.933 vs. 0.813, P<0.05), accuracy (0.79 vs. 0.70), precision (0.89 vs. 0.75), and F1-score (0.77 vs. 0.64). These gains indicate that multimodal feature integration and MLP’s nonlinear capacity capture clinically relevant patterns beyond simpler models.

Despite our study’s promising results in predicting CR after curative ablation for HCC, there are some limitations. First, the deep learning and radiomic models were constructed based on retrospective data; incorporating prospective data from more clinical trials would enhance the clinical evidence supporting our models. Second, this study should be regarded as a pilot investigation (32) rather than a definitive clinical tool. The cohort of 108 patients, while providing valuable preliminary data consistent with existing pilot studies on HCC radiomic prediction models (22, 40), requires validation in larger, independent populations before any clinical recommendations can be made. Third, while DCA suggests clinical utility across 15-45% thresholds, the optimal threshold requires prospective validation in multi-center cohorts (36). Fourth, the broad CR assessment window (1–12 months) precludes analysis of early versus late response; larger multi-center cohorts are needed for such temporal stratification (36, 41). Fifth, the high prevalence of HBV infection (84.7%) in our cohort reflects the epidemiological characteristics of HCC in East Asia, where HBV is the dominant etiology (42). However, this may limit the generalizability of our findings to regions where other risk factors predominate, such as HCV infection, alcohol-related liver disease, or non-alcoholic steatohepatitis (NASH) (43). The biological behavior of HCC and response to ablation therapy may differ across etiologies (1); for instance, HCV-related HCC is often associated with more advanced fibrosis and distinct tumor biology compared to HBV-related HCC (44). Therefore, our model’s performance in non-HBV populations remains to be validated. Additionally, our sample size was relatively small, and the study was limited to a single center. In the future, it will be essential to include a larger number of patients and data from multiple centers to address the sample size issue.

## 5 Conclusion

We developed a multimodal model based on a combination of features (Radiomics + Clinical + Deeplearning). Compared to single-modality features, the integration of multimodal features allows for a more comprehensive capture of patients’ pathophysiological information, enhancing the model’s discriminative ability. Among various classification algorithms, the MLP model demonstrated the best performance due to its effective nonlinear modeling capabilities and adaptability to complex data, achieving the highest AUC value under the multimodal feature combination. This underscores its superiority in predicting prognosis after HCC ablation. In this pilot study, we developed a multimodal model combining radiomics, deep learning, and clinical features to predict CR after HCC ablation. The MLP model achieved an AUC of 0.933, demonstrating promising predictive capability. However, the sensitivity of 0.50 for non-CR detection highlights a key limitation, indicating that this model should be considered a preliminary tool requiring external validation before clinical implementation (32). Future multi-center studies with larger cohorts, particularly enriched for non-CR cases, are essential to validate and potentially improve upon these findings (33, 42). Decision curve analysis confirmed the clinical utility within the threshold range of 15%-45%, albeit with limitations. With further validation, this approach may ultimately contribute to personalized treatment strategies for HCC patients undergoing ablation therapy.

## Data Availability

The original contributions presented in the study are included in the article/supplementary material. Further inquiries can be directed to the corresponding authors.
